# A systemic infection involved in lung, brain and spine caused by *Scedosporium apiospermum* species complex after near-drowning: a case report and literature review

**DOI:** 10.1186/s12879-023-08279-9

**Published:** 2024-03-21

**Authors:** Peng Yan, Junfeng Chen, Haodi Wang, Qi Jia, Jungang Xie, Guoxin Mo

**Affiliations:** 1 College of Pulmonary and Critical Care Medicine, Chinese People’s Liberation Army General Hospital, No. 17, Heishanhu Road, Haidian District, Beijing, 100091 China; 2 China Aerospace Science & Industry Corporation 731 Hospital, Beijing, China; 3 The Fifth People’s Hospital of Zunyi City, Zunyi City, Guizhou Province China; 4 Vision Medicals for Infection Diseases, Guangzhou, Guangdong China; 5 Pulmonary and Critical Care Medicine, Tongji Hospital Affiliated to Tongji Medical College, Huazhong University of Science and Technology, 1095 Jiefang Ave., Wuhan, Hubei China

**Keywords:** *Scedosporium apiospermum* species complex, Near-drowning, Systemtic infection, Metagenomic next-generation sequencing

## Abstract

*Scedosporium apiospermum* species complex are widely distributed fungi that can be found in a variety of polluted environments, including soil, sewage, and decaying vegetation. Those opportunistic pathogens with strong potential of invasion commonly affect immunosuppressed populations However, few cases of scedosporiosis are reported in immunocompetent individuals, who might be misdiagnosed, leading to a high mortality rate. Here, we reported an immunocompetent case of systemtic infection involved in lung, brain and spine, caused by *S. apiospermum* species complex (*S. apiospermum* and *S. boydii*). The patient was an elderly male with persistent fever and systemtic infection after near-drowning. In the two tertiary hospitals he visited, definite diagnosis was extremely difficult. After being admitted to our hospital, he was misdiagnosed as tuberculosis infection, before diagnosis of *S. apiospermum* species complex infection by the metagenomic next-generation sequencing. His symptoms were alleviated after voriconazole treatment. In the present case, the details associated with its course were reported and published studies on *Scedosporium* spp. infection were also reviewed, for a better understanding of this disease and reducing the misdiagnosis rate.

**Supplementary Information:**

The online version contains supplementary material available at 10.1186/s12879-023-08279-9.

## Introduction

*Scedosporium* spp. are widely distributed in soils of temperate climates, rather than tropical climates [1]. *Scedosporium boydii* (*Pseudallescheria boydii*) was once considered to be the sexual state of *Scedosporium apiospermum*. However, due to little difference in phylogenetic and clinical significance, both *S. apiospermum* and *S. boydii* can be described as “*Scedosporium apiospermum* species complex” [2].

At least 5 species of *Scedosporium* (*S. apiospermum*, *S. boydii*, *S. aurantiacum*, *S. dehoogii*, and *S. minutisporum*) can cause human infections [3], while *S. apiospermum* and *S. boydii* are fonud to be the two most common pathogens [4, 5]. They can cause systemic infections in immunosuppressed individuals, such as organ transplant recipients, and patients with hematological malignancies or receiving long-term glucocorticoid therapy [5]. In immunocompetent individuals, certain conditions, such as near-drowning or injuries may let *Scedosporium* cause therapy-refractory and life-threatening infections in the central nervous system (CNS) or lung, including respiratory symptoms, superficial infections, and severe invasive localized or disseminated mycoses [6, 7].

*Scedosporium* spp. are resistant to 5-flucytosine and amphotericin B, as well as to the first generation triazole drugs, fluconazole and itraconazole [5]. This species also shows a reduced susceptibility to echinocandins (particularly caspofungin and anidulafungin) and the triazole drug, isavuconazole. According to a global guideline for the diagnosis and management of rare mould infections, voriconazole represents the first-line treatment of *Scedosporium* infections [8].

The clinical manifestations of *Scedosporium* infection are complex, resulting in misdiagnosis. Here, we present the case of an immunocompetent patient with a systemic infection, which was found to be caused by *S. apiospermum* and *S. boydii* using metagenomic next-generation sequencing (mNGS). We also summarized literature reviews on *Scedosporium* infection in immunocompetent individuals.

## Case report

**Fig. 1:**
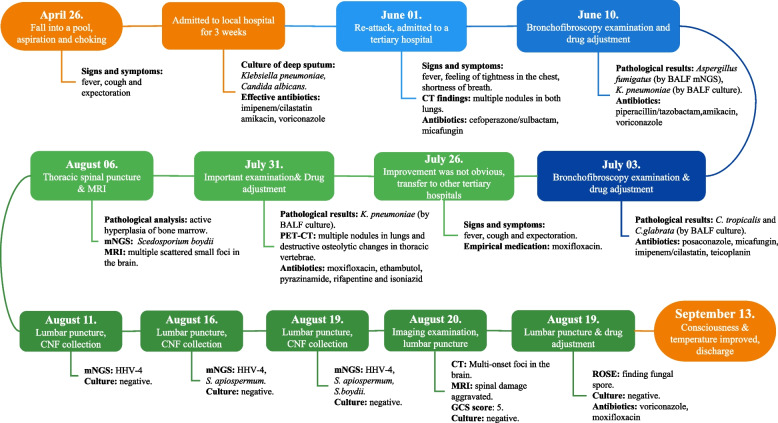
Timeline of patient journey. CT: Computed Tomography; mNGS: metagenomic next-generation sequencing; BALF: bronchoalveolar lavage fluid; PET: positron emission tomography; MRI: magnetic resonance imaging; GCS: Glasgow Coma Scale; ROSE: rapid on-site evaluation

**Fig. 2:**
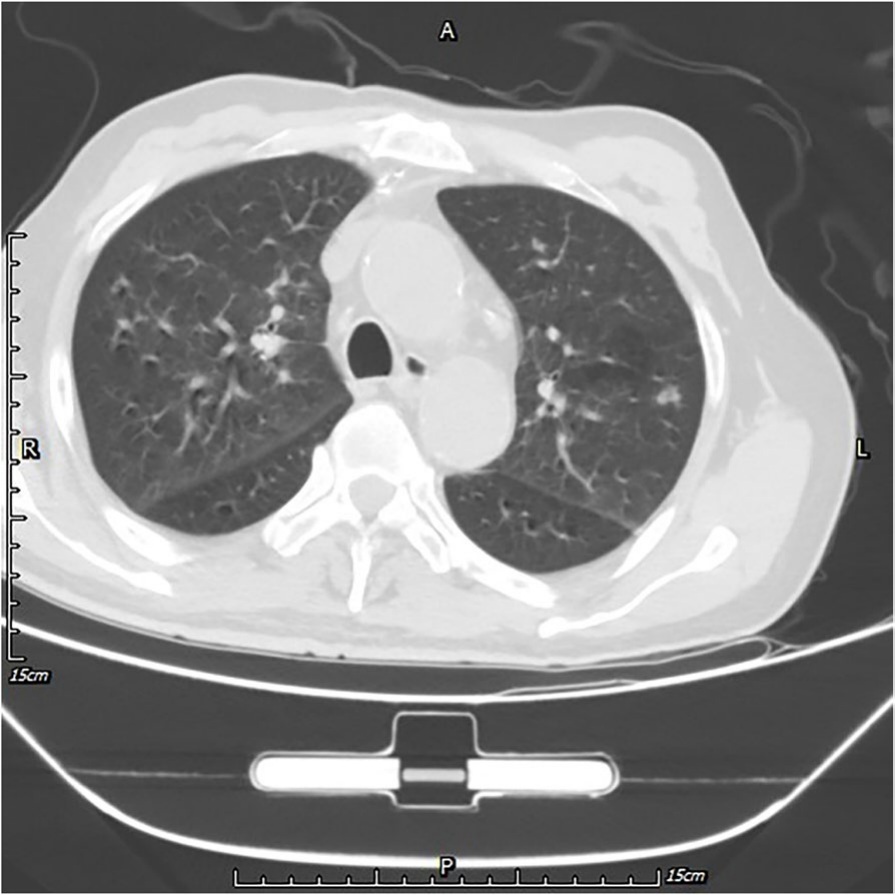
A lung CT (Computed Tomography) image showing multiple nodules in both lungs

The patient was a 70-year-old man who born and bred in a suburb of Wuhan, China, and both agriculture and industry exist in his surroundings. He had undergone lumbar disc surgery, rheumatoid arthritis, hypertension, and lacunar infarction. On April 26, 2021, he accidently fell into a pond (standing water) in a field (Figure S1). Aspiration and choking occurred spontaneously, and then fever, cough and expectoration started to appear. The highest body temperature reached 39.6 ℃, and the patient suffered from paroxysmal cough with a small amount of white sticky sputum, accompanied by shortness of breath, dizziness and transient loss of consciousness, without any symptoms of chest pain, hemoptysis, abdominal pain, hematemesis, or limb dysfunction. He was quickly admitted to a local hospital and was diagnosed with pulmonary infection. As *Klebsiella pneumoniae* and *Candida albicans* were successively cultured from deep sputum and the patient kept fever, multiple antibiotics were adopted and switched according to his clinical manifestation. With a drug combination of imipenem/cilastatin (500 mg/500 mg q.8.h. i.v.d), amikacin (400 mg q.12.h. i.v.d), and voriconazole (200 mg q.12.h. i.v.d) was adopted and lasted for 1 week, patient’s clinical manifestation was partially improved. He was discharged from the hospital on May 18, and did not adhere to the medication regimen since then. However, ten days after discharge, the patient developed fever again, with a body temperature of up to 39 ℃, accompanied by feeling of tightness in the chest and shortness of breath, without obvious cough or expectoration. Then, he visited Wuhan's Tongji Hospital where anti-infectives and oral antipyretic drugs were given in an emergency observation ward. Although the body temperature returned to normal, recurrent fever persisted, which led to a second hospitalization on June 1, 2021. Examination results showed increases in the values of various infection indicators, multiple nodules in both lungs from a computed tomography (CT) image (Figs. 1 and 2). Patient was empirically treated with cefoperazone/sulbactam (1,500 mg/1,500 mg q.12.h. i.v.d) and micafungin (100 mg q.d. i.v.d). Three days later, *Aspergillus fumigatus* was detected in bronchoalveolar lavage (BAL) fluid using mNGS (number of sequences: 15), and culture of BAL fluid by blood plate and China blue agar plat yielded *K. pneumoniae* again. However, the patient's blood culture (aerobic and anaerobic, up to 2 weeks), the (1,3)—β -D dextran test (G test), and the galactomannan test (GM test) were all negative. Therefore, micafungin was replaced by voriconazole (200 mg q.12.h. i.v.d), and cefoperazone and sulbactam was replaced by piperacillin/tazobactam (3,000 mg/375 mg q.6.h. i.v.d). Besides, amikacin (280 mg q.8.h. i.v.d) was added into the treatment prescription. However, the patient's symptoms was not significantly improved. On July 3, culture of BAL fluid was performed again and only yielded *C. tropicalis* and *C. glabrata*. The antifungal regimen was changed to posaconazole (400 mg q.12.h. p.o) combined with micafungin (100 mg q.d. i.v.d). Given his continuous high fever, piperacillin/tazobactam was replaced by imipenem/cilastatin (500 mg/500 mg q.8.h. i.v.d) combined with teicoplanin (400 mg q.d. i.v.d). After treatment, the symptoms of dizziness and shortness of breath disappeared, and the frequency of cough and expectoration was reduced.

**Fig. 3:**
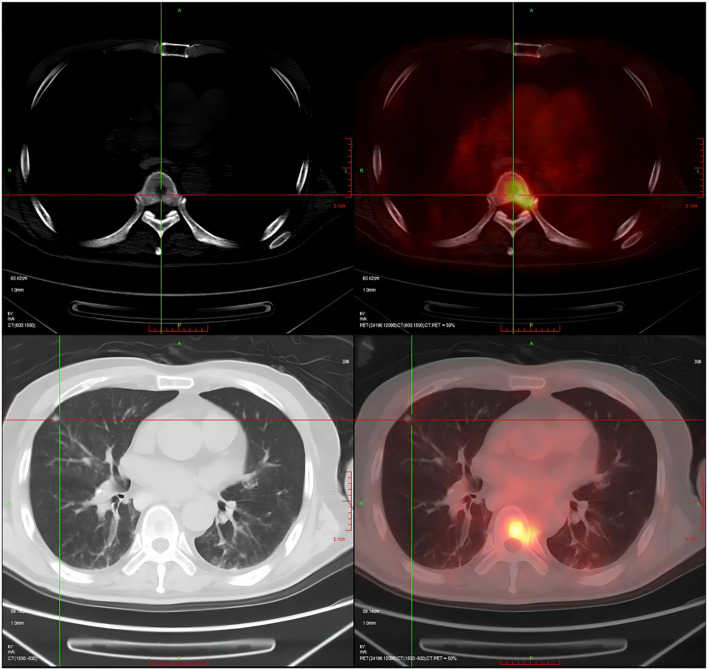
PET/CT (Positron emission tomography computed tomography) imaging revealed multiple nodules and streaks in both lungs and destructive osteolytic changes between T6 and T7, implying infectious lesions. Large bullae and calcification nodules were found in the upper lobe of the left lung

**Fig. 4:**
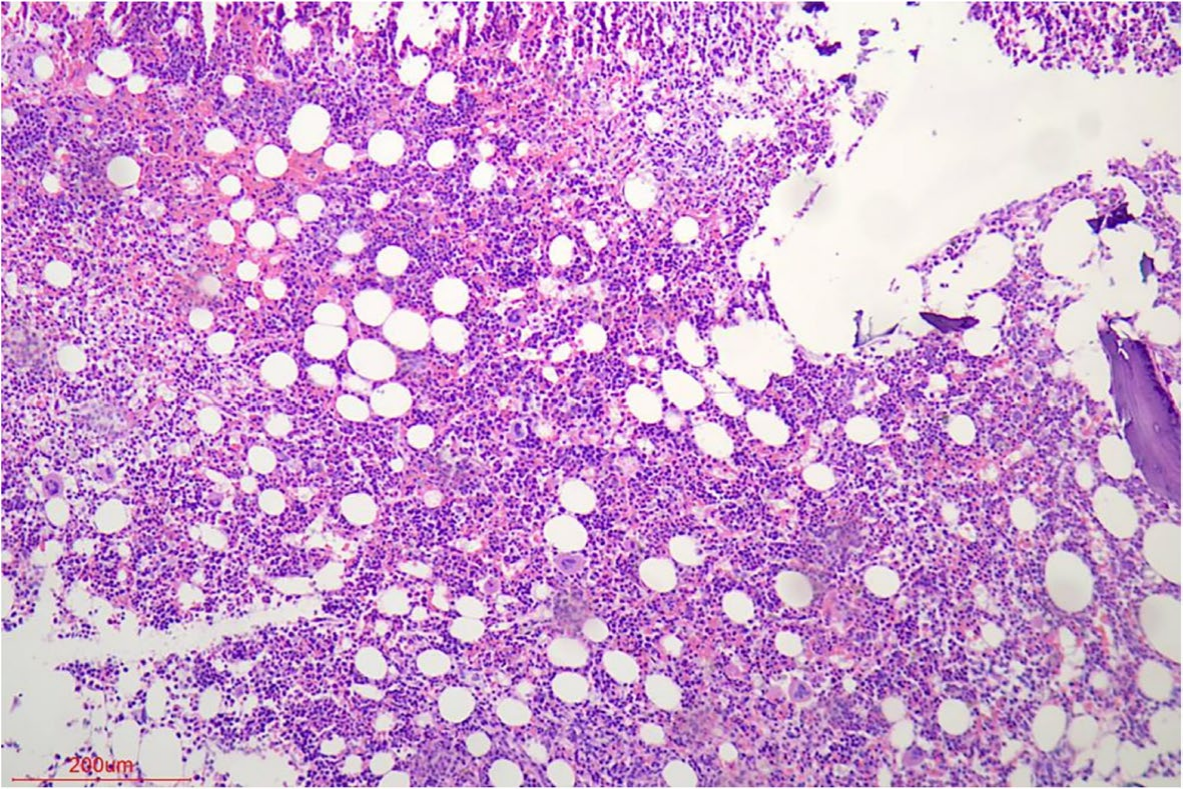
The pathological analysis of bone tissues showed excessive bone marrow tissues in trabecular bones and implied active hyperplasia of bone marrow

**Fig. 5:**
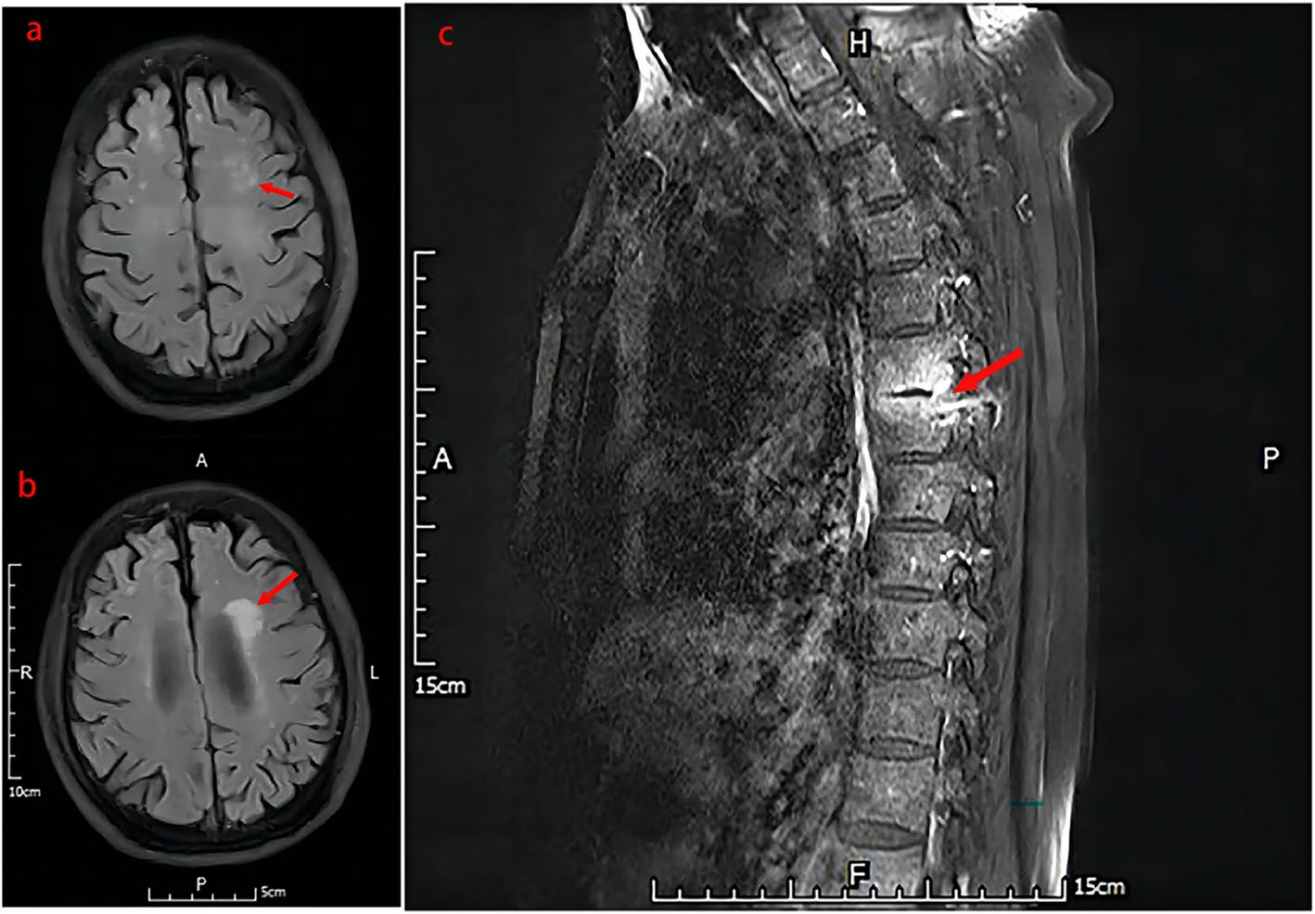
Contrast-enhanced MRI: The left temporal lobe (**a**) and lateral paraventricular (**b**) demonstrated abnormal signal and enhancement, considering infectious lesions. The thoracic vertebrae (**c**) demonstrated abnormal signals unevenly enhanced at T6-T7 accompanied by slight patchy enhancement of the swelling soft tissues in the vicinity

Unfortunately, the patient still experienced recurrent episodes of fever, and had a significantly reduced ability to perform daily activities with fatigue leaving him bedridden. Due to worsening symptoms of fever, cough and expectoration lasting for 3 days, the patient was eventually transferred to our hospital on July 26, 2021. Antipyretic drugs were given to temporarily control the body temperature which had been up to 39 °C (sustained fever), but the symptom of cough with a small amount of white sticky sputum remained. Meanwhile, symptoms of chest pain, night sweats, hemoptysis, palpitation, wheezing, abdominal pain, and diarrhea were not observed. BAL fluid cultured yielded *K. pneumoniae* again, but by Sabouraud dextrose agar (SAD) plat yielded negative. Thus, moxifloxacin (400 mg q.d. i.v.d) was empirically applied to treat infections before diagnosis of the specific pathogen. On the 3^rd^ day of admission, positron emission tomography (PET)/CT imaging (Fig. 3) revealed multiple nodules and streaks in both lungs and destructive osteolytic changes between T6 and T7, implying infectious lesions. Based on the consultation results from the Tuberculosis Department, the empirical anti-tuberculosis treatment was introduced, consisting of ethambutol, pyrazinamide, rifapentine and isoniazid. Seven days after admission, a thoracic spinal puncture at T6-T7 was performed with tissues sampled for examination. The pathological analysis of bone tissues (Fig. 4) showed excessive bone marrow tissues in trabecular bones, indicating active hyperplasia of bone marrow, and *S. boydii* (number of sequence: 1) was detected by mNGS. As the number of sequences was very low, *S. boydii* was not considered as a pathogen, but as a pollutant. On the 10^th^ day of admission, contrast-enhanced magnetic resonance imaging (MRI) was carried out for brain examination and result indicated multiple scattered small foci of signal abnormality with enhancement in the brain, multiple lacunar infarcts and ischemic foci (Fig. 5), according to which the patient was initially diagnosed with infectious disease, tuberculous meningitis in particular. This hypothesis was further supported by persistent fever and systemtic dysfunction. For further confirmation of *Mycobacterium tuberculosis* infection, a series of tests were conducted, including the purified protein derivative (PPD) test, contrast-enhanced spinal MRI, and genetic testing, but all results turned out to be negative. Subsequently, lumbar puncture was performed on the 14^th^ day of admission and cerebrospinal fluid (CSF) was then collected for examination. CSF examination showed a significant increases of total cell count (360 × 10^6^/L), white blood cell counts (189 × 10^6^/L), and trace protein (2.16 g/L), while concentrations of chlorine and glucose were at normal levels; Human herpes virus infection indicated by mNGS (Table 1); culture yielded negative. As a result, the patient was maintained with moxifloxacin treatment and quadruple therapy for tuberculosis. Twenty days after admission, lumbar puncture and CSF examination were repeated. It’s cytologic and biochemical results showed no big change than before, however, only 1 sequence of *S. apiospermum* was detected at this time by mNGS. CSF culture still yielded negative. Subsequently, the patient had his third lumbar puncture and CSF examination on the 23^rd^ day of admission, with the reports showed the total cell count was nearly 11 times increased compared to base line, but the WBC count and trace protein decreased. mNGS porformed again and Human herpes virus-4 (number of sequences: 5), *S. boydii* (number of sequences: 2) and *S. apiospermum* (number of sequence: 1) were determined. On the 24^th^ day of admission, the patient was found unresponsive to speech, no eye-opening and no speech during examination, but responses to painful stimuli with a Glasgow Coma Scale (GCS) score of 5. From a new cranial CT scan, multiple low-density lesions, mild hydrocephalus, and multiple lacunar infarcts in the brain were observed. Furthermore, contrast-enhanced MRI of the thoracic vertebrae demonstrated abnormal signals unevenly enhanced at T6-T7 accompanied by slight patchy enhancement of the swelling soft tissues in the vicinity, and hyperintense signals at the intervertebral disc between T9 and T10 (Fig. 5). Then, lumbar puncture and CSF examinations were carried out for the fourth time, and it’s result showed a decreased number of total cell count (621 × 10^6^/L) but an increased number of WBC count (594 × 10^6^/L); the biochemical results and number of pathogen sequence obtained using mNGS Microbiologic rapid on-site evaluation of CSF revealed a fungal spore, but culture still yielded negative.

**Table 1:** Results of patient’s CSF examinations in admission

Date	Total cells count (× 10^6^/L)	WBC count (× 10^6^/L)	Chlorine (mmol/L)	Glucose (mmol/L)	Trace protein (g/L)	Adenosine deaminase (U/L)	Lactate dehydrogenase (U/L)	Number of sequence
11/8	360	189	108.91	1.48	2.16	-	-	HHV-4: 437
16/8	360	189	120.55	2.25	1.82	3.3	81	*S. apiospermum*: 1
19/8	4100	110	124.54	3.54	1.36	1.8	57	HHV-4: 5; *S. apiospermum*: 1; *S.boydii*: 2
20/8	621	594	114.06	2.05	1.68	2.1	55	-
23/8	3900	300	125.54	2.98	1.76	2.1	79	Negative
27/8	117	45	127.67	3.72	0.97	2.0	51	Negative
13/9	22	22	117.95	7.46	1.06	1.4	33	Negative

Character of 7 CSF samples were clear and colorless and Tuberculosis related examinations were always negative

*S. apiospermum* *Scedosporium apiospermum*, *S.boydii* *Scedosporium boydii*, *HHV* Human herpes virus

Due to the history of near-drowning, it was suspected that this case of systemtic infection (lung, brain and spine) was caused by *S. apiospermum* species complex, and antifungal therapy with voriconazole (200 mg q.12.h. i.v.d) was thereafter included to the existing regimen. One week after voriconazole treatment, the body temperature returned to normal, but the overall cognitive ability was still poor. Though the patient was able to remember his own name, he could not perform simple calculations and had no memory of being admitted to the hospital. With the treatment continued, the patient's state of consciousness was gradually improved, as evidenced by the ability to perform addition and subtraction within 10 and recognize some of his families after half a month of treatment. The patient's cognitive ability almost returned to normal 3 weeks later when the patient could recognize his families, remember his home address, perform multiplications and get out of bed. After discharge, voriconazole was changed to oral administration and the entire treatment of scedosporiosis continued for 6 months. Follow-up at 8 months after discharge, CNF mNGS detected no microorganisms. The patient had sequelae of slow response and no other special discomfort.

## Review and discussion

The incidence rate of scedosporiosis has been increasing in recent years. As an emerging fungal pathogen, *Scedosporium* spp. is receiving an increasing attention. We searched PubMed to find articles on cases with *Scedosporium* (including “*Pseudallescheria/Scedosporium* complex”, except “*Lomentospora prolificans”*) deep infection in immunocompetent individuals, which were published between 1982 and 2022 (Table 2).

**Table 2:** Case reports with Scedosporium in immunocompetent individuals between 1982 and 2022

No	Reference	Patient (Age/Sex)	Country	Initial event	Risk factors	Signs and Symptoms	Period after the initial event	Affected parts	Identification of Scedosporium	Method of identification	Antifungal treatment	Treatment Duration	Outcome
1	Hung LH et al., 1993 [9]	32/M	USA	Trauma	None	Pain and swelling in the left knee	6 years	Knee and proximal tibia	*S. boydii*	Culture	KETO	Not mentioned	Improved
2	Rüchel R et al., 1995 [10]	21/M	Germany	Near-drowning	None	Fever, drowsiness, spastic paralysis	c. 1 week	Brain, heart	*S. apiospermum*	Culture and Microscopy	Flucytosine + FLU	26 days	Death
3	Khurshid A et al., 1999 [11]	61/F	USA	None	Bullous emphysema	Weight loss, night sweats, cough, fever, and hemoptysis	Not applicable	Lung,heart, liver, spleen, kidney, pancreas, and brain	*S. boydii*	Culture	AmB	Unknown	Death
4	Poza G et al., 2000 [12]	24/M	Spain	Surgical operation	None	Headache, back pain	c. 1 month	CNS	*S. boydii*	Culture	VCZ	1 year	Improved
5	Tirado-Miranda R et al., 2001 [13]	32/M	Spain	Traffic accident	None	Fever, swelling and pain in the knee	53 days	Right knee	*S. aurantiacum*	Culture	1)AmB 2)ITRA	Not mentioned	Improved
6	Kiraz N et al., 2001 [14]	24/F	Turkey	None	None	Enlarged cervical lymph nodes	Not applicable	Lymph nodes	*S. apiospermum*	Culture and Microscopy	ITRA	1 year	Improved
7	Levine NB et al., 2002 [15]	52/M	USA	None	None	Thoracolumbar spinal pain, hemoptysis	Not applicable	Spinal column	*S. apiospermum*	Culture	ITRA	Not mentioned	Death
8	Farina C et al., 2002 [16]	23/M	Italy	Traffic accident	None	Purulent secretions	3 days	Skull	*S. apiospermum*	Culture	AmB	21 days	Improved
9	Chakraborty A et al., 2005 [17]	1.5/M	UK	Near-drowning	None	Low-grade fever, unconsciousness, grand mal seizures	3 months	Brain	*S. apiospermum*	Culture	1)ITRA 2)AmB 3)VCZ	c. 18 months	Improved
10	Kooijman CM et al., 2007 [18]	36/M	The Netherlands	Trauma	None	Fistula and abscess	c. 11 weeks	Femur	*S. aurantiacum*	Culture	VCZ	8 weeks	Improved
11	Leechawengwongs M et al., 2007 [19]	21/M	Thailand	Traffic accident, near-drowning	None	Headache, unconsciousness	14 days	Brain	*S. apiospermum*	Culture	1)AmB + CASPO 2)VCZ	23 months	Improved
12	Stripeli F et al., 2009 [20]	10/F	Greece	Trauma	None	Knee swelling and pain	c. 2 months	Left knee	*S. apiospermum*	Culture and Sequencing	1)AmB 2)VCZ	6 moths	Improved
13	Al-Jehani H et al., 2010 [21]	33/M	Canada	Extra-Corporeal Membrane Oxygenation	Neutropenia	Fever, decreased movement, partial seizure	c. 14 days	Brain	*S. apiospermum*	Culture	VCZ	1 week	Death
14	Hell M et al., 2011 [22]	16/M	Austria	Trauma	None	Soft tissue healing defect, persisting fistula	c. 3 weeks	Bone, muscle	*S. apiospermum*	Culture	VCZ	c. 6 months	Improved
15	Cumbo-Nacheli G et al., 2012 [23]	62/F	USA	None	Mycobacterium avium complex infection history	Fever, dyspnea, cough, and worsening pulmonary nodules	Not applicable	Lung	*S. boydii*	Pathological examination	VCZ	Not mentioned	Improved
16	Angelini A, Drago G et al., 2013 [24]	27/F	Italy	Suffered tsunami	None	Pain in the knee	2 years	Left knee	*S. apiospermum*	Culture and Sequencing	1)VCZ 2)POSA + TERB	> 1 year	Improved
17	Wilson HL et al., 2013 [25]	69/M	Australia	None	Silicosis, COPD	Headache, fever, lethargy, nausea and vomiting	Not applicable	Brain	*S. apiospermum*	Culture, Microscopy and Sequencing	1)ABLC 2)VCZ 3)CASPO	c. 2 months	Death
18	Cruysmans C et al., 2015 [26]	7/M	UK	Trauma	None	low-grade fever, lethargy, weakness of lower limb	5 moths	Endorachis	*S. apiospermum*	Culture	VCZ	1 year	Improved
19	Rahman FU et al., 2016 [27]	40/M	Pakistan	None	Pulmonary TB history	Cough, hemopty	Not applicable	Lung	*S. apiospermum*	Culture and Microscopy	VCZ	4 moths	Improved
20	Dinh A et al., 2018 [28]	57/M	France	None	None	Iterative fractures	Not applicable	Tibial pseudarthrosis	*S. apiospermum*	Culture and MALDI-TOF	VCZ	6 moths	Improved
21	Tan SYL et al., 2020 [29]	39/M	China	Percutaneous driveline tugged	Left ventricular assist devices equipping	Fever, local pustular lesion	c. 2 months	Blood stream	*S. apiospermum*	Culture and Sequencing	Not able to take	Not applicable	Death
22	Jabr R et al., 2020 [30]	72/M	USA	Peripherally inserted central catheter placement	Pulmonary arterial hypertension	Fever, intermittent hemoptysis, worsening shortness of breath	c. 1 month	Blood stream	*S. apiospermum*	Culture	1)VCZ 2)ABLC 3)TERB	7 months	Improved
23	Liu W et al., 2020 [31]	44/M	China	None	None	Hemoptysis	Not applicable	Lung	*S. apiospermum*	Culture and Microscopy	VCZ	c. 11 months	Improved
24	Mir WAY et al., 2021 [32]	83/F	USA	None	Chronic atrial fibrillation, COPD	Shortness of breath, cough with blood-tinged sputum, fatigue	Not applicable	Lung	*S. apiospermum*	Culture	VCZ	6 moths	Improved
25	Ghasemian R et al., 2021 [33]	67/F	Iran	Near-drowning	None	Fever, respiratory distress	7 days	Lung	*S. aurantiacum*	Culture and Sequencing	1)VCZ 2)ABLC	6 days	Death
26	Song Y et al., 2022 [34]	56/M	China	Inhalation of Biogas	None	Nausea, vomiting, haemoptysis, fever	10 days	Lung	*S. apiospermum*	Culture and MALDI-TOF	1)VCZ 2)ABLC	230 days	Improved
27	Shi XW et al., 2022 [35]	60/M	China	None	None	Lumbosacral pain, stooped back, restricted walking	Not applicable	Lumbar vertebra	*S. apiospermum*	Culture	VCZ	6 moths	Improved
28	This study	70/M	China	Near-drowning	None	Fever, chest tightness, shortness of breath, dizziness	c. 1 month	Lung, brain and spine	*S. apiospermum, S. boydii*	mNGS	VCZ	6 months	Improved

*S. apiospermum*: *Scedosporium apiospermum, S. aurantiacum*: *Scedosporium aurantiacum, S.boydii*: *Scedosporium boydii*

*M* Male, *F* Female, *ABLC* Amphotericin B (Lipid Complex), *AmB* Amphotericin B deoxycholate, *CASPO* Caspofungin, *FLU* Fluconazole, *ITRA* Itraconazole, *KETO* Ketoconazole, *POSA* Posaconazole, *TERB* Terbinafine, *VCZ* Voriconazole

Among the 28 published studies, there were 21 males and 7 females infected by *Scedosporium apiospermum* species complex. Age of those patients ranged from 1.5 to 83 years old. Besides, 15 patients (53.6%) suffered trauma or near-drowing, and 3 patients (10.7%) received invasive medical treatment, while any initial events of 10 patients (35.7%) were not reported. The interval of 18 cases from the initial event to onset ranged from 3 days to 6 years.

The most common infection sites were bone, muscle, and joint (11 cases, 39.3%) followed by CNS (include brain and endorachis, 9 cases, 32.1%) and lung (8 cases, 28.6%). Most importantly, 4 out of 9 cases with CNS infection had a history of near-drowing. Fever was the most common systemic symptom (13 cases, 46.4%), which was often associated with infection dissemination. Clinical manifestation of focal infections mainly included local pain, swelling, and dysfuncion, while fever was found in few cases.

*S. apiospermum* was found to be the causative pathogen in most of patients (21 cases, 75%), follow by *S. boydii* (5 cases, 17.9%) and *S. aurantiacum* (3 cases, 10.7%). Among 27 patients receiving anti-fungal drugs, voriconazole treatment was performed on 20 cases (71.4%) and most of them (17 out of 20, 85%) had improved outcome, while more than half of patients (4 out of 7, 57.1%) without voriconazole treatment had poor prognosis.

Among 18 cured cases, treatment duration ranged from 21 days to 23 months. The length of the treatment duration was related to infection sites. Patients with CNS infection need the longest treatment duration (428 days ± 174 days), followed by infections of cardiovascular and lymph nodes (313 days ± 73 days), pulmonary infection (215 days ± 77 days), and infections of bone, muscle, and joint (166 days ± 102 days). The clinical outcome of disseminated or CNS infection is dismal. Previous studies have shown that the mortality rate can reach up to 65%-100%, once *Scedosporium* disseminates systematically or invades the brain [36].

Culture has always been gold standard of fungal infection diagnosis, and microscopy and molecular biology methods such as sequencing or Matrix-Assisted Laser Desorption/ Ionization Time of Flight Mass Spectrometry (MALDI-TOF–MS) are often adopted after successful cultivation. However, due to the low load of fungi in the CNS, culture is insensitive diagnostic tool. In our case, blood and CSF samples were used to isolate pathogens many times. However, we cannot successfully isolate any fungai. Fortunately, using mNGS, we successfully detected *Scedosporium* from bone marrowand CSF specimens, which was diagnosed as pathogen.

NGS is an emerging microbiological sequencing diagnostic approach which has advantages of culture-independent, short turnaround time, and high efficiency in cataloging and recognizing pathogens [37]. In this case, although the number of reads detected was low, mNGS results of samples from multiple sites were positive, according to which doctors made a preliminary clinical diagnosis of scedosporiosis. Fortunately, the patient had a good response to the subsequent treatment with voriconazole, which also confirmed the correctness of the clinical diagnosis. However, our case report has limitations, including lack of classical evidence of fungal culture, drug susceptibility testing, and precise species identification. Future study should focus on howto improve the accuracy and specificity of mNGS in fungal pathogen detection to provide more information on fungal drug resistance.

## Conclusion

Collectively, scedosporiosis is a rare and challenging illness to diagnose. The pathogens should be confirmed as soon as feasible for a patient who has risk factors, such as being close to drowning, by microbiological analysis and histological inspection of specimens taken from the damaged tissues, along with clinical manifestations and imaging data. Appropriate treatment should be provided quickly so as to reduce the mortality rate.

## Patients consent

Patients provided informed consent for the publication of the cases.

### Supplementary Information

**Additional file 1:** **Figure S1.** The pond the patient accidentally fell into.

## Data Availability

Sequencing data were deposited to the National Genomics Data Center under accession numbers PRJCA014995 and CRA009845. The datasets used and/or analyzed during the current study are available from the corresponding author on reasonable request.
