# Supplementary figures and images for: A systemic infection involved in lung, brain and spine caused by Scedosporium apiospermum species complex after near-drowning: a case report and literature review
Source: BMC Infect Dis. 2024 Mar 21;24:342. doi: 10.1186/s12879-023-08279-9 (PMC10956195; doi:10.1186/s12879-023-08279-9)

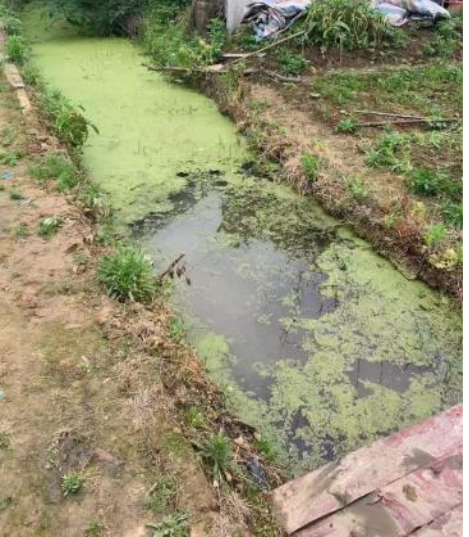


Figure S1. The pond the patient accidentally fell into.

Supplement: Supplementary file 1 — Additional file 1: Figure S1. The pond the patient accidentally fell into. [file 12879_2023_8279_MOESM1_ESM.docx]
